# Dynamic alteration of serum testosterone with aging: a cross-sectional study from Shanghai, China

**DOI:** 10.1186/s12958-015-0107-z

**Published:** 2015-09-30

**Authors:** Zhangshun Liu, Jie Liu, Xiaohong Shi, Lihong Wang, Yan Yang, Minfang Tao

**Affiliations:** Department of Reproductive Medicine Center, Shanghai Jiao Tong University Affiliated Sixth People’s Hospital, No. 600 Yishan Road, Shanghai, 200233 China

**Keywords:** Adult male, Androgen, Serum testosterone

## Abstract

**Background:**

Level of the testosterone in a man’s life span is very important. Studies on the serum testosterone concentrations in different age groups of healthy men were controversial. The aim of this study was to investigate dynamic changes of serum reproductive hormones with aging in healthy Chinese male and to compare its correlation with age.

**Methods:**

Total of 1,093 healthy Chinese men from Shanghai aged from 20 to 87  years old was enrolled in the study. Concentrations of serum total testosterone (T), luteinizing hormone (LH) and sex hormone binding globulin (SHBG) were quantified by EIA. Testosterone secretion index (TSI) and free testosterone index (FTI) were then calculated. Data were analyzed by SPSS program. Non-parametric tests and univariate linear regression analyses were used.

**Results:**

The 1,039 male participants were grouped into 12 groups by 5-year apart for each group. Significant differences in T, LH, SHBG, FTI and TSI were found between the 12 different age groups. Average of serum total T was 15.36 ± 4.86 nmol/L; LH was 4.76 ± 2.76 IU/L, SHBG was 32.61 ± 17.24 nmol/L. Compared to age 20 ~ 24 group, serum T level of age 35 ~ 39, 40 ~ 44, 45 ~ 49, 50 ~ 54, and 55 ~ 59 was significantly decreased (*p* < 0.05). Intriguingly, however, serum T level of age 60 or older did not significantly reduced compared to the age of 20 ~ 24 group. Serum LH and SHBG were positively correlated with aging (*p* <0.01), while TSI and FTI were negatively correlated with aging (*p* <0.01). In addition, BMI was negatively and significantly correlated with levels of T (*r* = −0.585, *p* < 0.001), LH (*r* = −0.090, *p* < 0.001), SHBG (r = − 1.817, *p* < 0.001), and TSI (*r* = − 0.104, *p* < 0.001), but positively and significantly correlated with FTI level (*r* = 0.011, *p* < 0.001).

**Conclusion:**

Serum total testosterone fluctuated with aging in adult men, and FTI and TSI decreased gradually with aging. While age was not significantly correlated with T level, BMI was significantly and negatively correlated with T level, suggesting body weight may affect testosterone level.

## Introduction

Testosterone plays key roles in developing secondary sexual characteristics, supporting spermatogenesis and regulating libido in the male [1]. Low-serum testosterone has been associated with numerous age-related adverse health conditions including abdominal obesity, diabetes, and pre-diabetic states (such as insulin resistance, impaired glucose tolerance, and metabolic syndrome), dyslipidemia, low bone and muscle mass, impaired sexual function, depressed mood, frailty, and decreased quality of life [2, 3]. In this regard, obesity may contribute to low total testosterone levels that can be explained at least partially by lower sex hormone-binding globulin (SHBG) in obese men [4, 5].

The incidence of testosterone deficiency (TD) increased with age due to decline in testosterone production after age 30 [6, 7]. Testosterone supplementation therapy (TST), therefore, has now become one of the most widely used medications in aged men with the increasing proportion of the population > 65 years of age and the increasing incidence of medical comorbidities associated with low testosterone, such as diabetes, metabolic syndrome, cardiovascular disease and erection dysfunction [8, 9]. The incidence of TD in healthy, middle-aged men has been reported as high as 6 %, and men in the seventies of age have mean plasma testosterone levels 35 % lower than that of young men [10–12]. Despite increasing incidence of TD, diagnosis and treatment of TD remains challenge for clinician because symptoms of males with TD are nonspecific and variable, and thus, only 12 % of clinically symptomatic, hypogonadal males are successfully treated with TST [10, 13].

While level of the testosterone in men’s life span is very important, decline of serum testosterone level in healthy men may vary depending on ethnics, environment, and life style such as food. In this content, it has been reported that total serum testosterone level was significantly higher in Caucasians (aged 22–27 yr) from the USA than that in Chinese (aged 24–39 yr) living in Beijing, China [14]. Furthermore, serum level of total testosterone in Chinese men from Pennsylvania (aged 20–37 yr) was also significantly higher than that of Chinese living in Beijing, China, although it was lower compared to that of Caucasians [14]. Similarly, Chinese living in China had significantly lower serum SHBG level compared to Chinese living in the USA [14].

Testosterone is produced by Leydig cells, which are terminally differentiated cells, and is regulated by luteinizing hormone (LH), which is synthesized and produced by the pituitary gland. Role of LH in regulating serum testosterone concentration in human being remains to be defined. In this regard, in the rodent aging models of Sprague–Dawley rats, it was found that aging lead to decreased LH levels that resulted in reduced stimulation of the Leydig cells [15]. In contrast to the rodent, it was found that serum LH levels rose moderately or did not change at all in older men even though declines in serum testosterone occurred [10, 16, 17]. In the current study, therefore, we investigated dynamic alteration of serum total testosterone (T), luteinizing hormone (LH) and sex hormone binding globulin (SHBG) in 1,093 healthy Chinese men whose age was from 20 to 87 years old. Testosterone secretion index (TSI) and free testosterone index (FTI) were also calculated.

## Materials and methods

### Patients

The current study was designed as a cross-sectional and single-center population-based observational study on endocrine status in adult men. Total 1,513 men were interviewed and 1,093 of them were finally enrolled in the current study. The inclusion criteria were: 1). At least 20 years old male; 2). No self-report of chronic illness (diabetes, high blood pressure, heart disease, cancer); 3). No current prescription medication known to affect the hypothalamic-pituitary-gonadal (HPG) axis (including testosterone, anti-androgen, glucocorticoid, opiate, antiepileptic, antipsychotics, 5-α reductase inhibitors, or aromatase inhibitors); 4). No genital system surgery.

### Consent to publish

This study was approved by The Institutional Review Board of Ethics Committee of Shanghai Jiao Tong University Affiliated Sixth People's Hospital. All participants received written and oral information prior to giving written consent, and the study was performed in accordance with the Helsinki II declaration.

### Sample collection

Blood sample was collected by venipuncture between 8 and 10 AM after an overnight fast of at least 8 h. After centrifugation, serum was harvested and stored at −80 °C until being processed.

### Assays

Chemiluminescence method, a commercially available diagnostic reagent kit and chemiluminescent analyzer, was used to measure serum T and LH (Beckman Coulter Inc, USA). Sensitivity of the assay was 0.01 nmol/L for testosterone and 0.2 IU/L for LH; the intra-assay coefficient of variation (CV) was 3.93 % and 3.8 %, and the inter-assay CV was 7.08 % and 6.4 %.

Serum SHBG level was determined by Two-step Enzyme ImmunoAssay (Beckman Coulter Inc, USA). The sensitivity was 0.2 nmol/L, intra-assay CV was 5.3 %, and inter-assay CV was <7 %. Diphosphoglycerate, triglyceride, and total cholesterol were also measured.

Testosterone secretion index (TSI) was calculated as testosterone divided by LH. Free testosterone index (FTI) was calculated as testosterone divided by SHBG. Body mass index was calculated as patient’s weight (kg) divided by squared height (m^2^).

### Statistical analysis

Data was verified and input into SPSS database, and was analyzed with SPSS 21.0 statistical package. Non-parametric tests were conducted in that all measured or calculated parameters were in abnormal distribution after normality test, even after logarithmic transformation. The median was considered to represent average level of the measured value, the 10th percentile and the 90th percentile represented the lower and upper limit of the reference value. Mann-Whitney U test was used to compare the sex steroids as well as correlation parameters between the 12 age groups of each decade.

The association between sex steroids and age was assessed by univariate linear regression analyses. Calculations were not adjusted for multiple comparisons as variables were correlated and all the tests were performed in an exploratory manner. Calculations were performed using SPSS v21.0 with *p* values < 0.05 considered significant.

## Results

### Characteristics of the study population

Total of 1,093 healthy males, who were 20 to 87 years old, participated in the current study. They were divided into 12 groups by 5-year of age apart. Of them, 20 participants were 20 ~ 24 years old; 185 were 25 ~ 29; 146 were 30 ~ 34; 101 were 35 ~ 39; 94 were 40 ~ 44; 65 were 45 ~ 49; 63 were 50 ~ 54; 87 were 55 ~ 59; 116 were 60 ~ 64; 92 were 65 ~ 69; 48 were 70 ~ 74; and 56 participants were 75 or older.

### Dynamic alteration of serum reproductive hormones

Dynamic alteration of serum levels of T, LH, SHBG, and calculated FTI and TSI was presented in Table 1. Average of total serum T was 15.36 ± 4.86 nmol/L (95 % CI: 15.07 ~ 15.65, *n* = 1093); LH was 4.76 ± 2.76 IU/L (95 % CI: 4.60 ~ 4.93), SHBG was 32.61 ± 17.24 nmol/L (95 % CI: 31.49 ~ 33.72), FTI was 0.55 ± 0.24 % (95 % CI: 0.53 ~ 0.56), and TSI was 3.98 ± 2.01 nmol/IU (95 % CI: 3.86 ~ 4.10).

**Table 1:** Serum levels of hormones in chinese male at age 20 to 87

Age group	Number	Serum level, mean ± SD (95 % CI)				
		T (nmol/L)	LH (IU/L)	SHBG (nmol/L)	FTI( % )	TSI (nmol/IU)
20~	40	17.31 ± 4.95 (15.73,18.89)	4.06 ± 1.73 (3.51,4.61)	25.91 ± 10.39 (22.59,29.23)	0.73 ± 0.21 (0.66,0.80)	4.79 ± 1.91 (4.18,5.40)
25~	185	16.18 ± 4.57 (15.52,16.85)	3.97 ± 1.73 (3.72,4.22)	25.44 ± 11.29 (23.80,27.08)	0.71 ± 0.27 (0.67,0.75)	4.65 ± 1.87 (4.38,4.92)
30~	146	15.46 ± 4.59 (14.71,16.21)	3.76 ± 1.53 (3.51,4.01)	28.69 ± 15.74 (26.12,31.27)	0.62 ± 0.22* (0.59,0.66)	4.67 ± 2.18 (4.31,5.03)
35~	101	14.40 ± 4.33* (13.54,15.25)	3.84 ± 1.35 (3.57,4.11)	26.77 ± 12.08 (24.39,29.16)	0.59 ± 0.20* (0.55,0.63)	4.17 ± 1.87 (3.80,4.54)
40~	94	14.33 ± 4.91* (13.33,15.34)	3.79 ± 1.66 (3.45,4.13)	30.49 ± 14.11 (27.60,33.38)	0.51 ± 0.16* (0.48,0.55)	4.44 ± 2.40 (3.95,4.93)
45~	65	14.22 ± 4.17* (13.18,15.25)	4.02 ± 1.95 (3.54,4.51)	32.90 ± 14.28* (29.36,36.44)	0.47 ± 0.13* (0.44,0.50)	4.19 ± 1.93 (3.71,4.66)
50~	63	14.19 ± 3.93* (13.20,15.18)	4.28 ± 1.72 (3.85,4.71)	34.01 ± 13.66* (30.57,37.46)	0.46 ± 0.16* (0.42,0.50)	3.83 ± 1.78* (3.38,4.28)
55~	87	14.73 ± 4.87* (13.69,15.77)	4.63 ± 2.67 (4.06,5.20)	38.18 ± 17.07* (34.54,41.82)	0.43 ± 0.14* (0.40,0.46)	3.78 ± 1.62* (3.43,4.12)
60~	116	15.32 ± 4.81 (14.43,16.20)	6.10 ± 3.56* (5.44,6.75)	43.27 ± 17.44* (38.09,48.45)	0.35 ± 0.10* (0.32,0.38)	3.31 ± 1.90* (2.96,3.66)
65~	92	16.18 ± 5.49 (15.05,17.32)	6.33 ± 3.06* (5.69,6.96)	45.19 ± 13.19* (40.36,50.03)	0.34 ± 0.06* (0.31,0.36)	3.08 ± 1.68* (2.73,3.43)
70~	48	16.36 ± 6.23 (14.55,18.17)	6.87 ± 3.86* (5.75,7.99)	50.64 ± 25.84* (39.47,61.81)	0.34 ± 0.09* (0.30,0.38)	2.95 ± 1.58* (2.49,3.41)
75~	56	15.96 ± 5.56 (14.47,17.45)	8.32 ± 4.19* (7.19,9.44)	60.64 ± 26.05* (52.07,69.20)	0.26 ± 0.09* (0.23,0.29)	2.31 ± 1.14* (2.00,2.61)
Total	1093	15.36 ± 4.86 (15.07,15.65)	4.76 ± 2.76* (4.60,4.93)	32.61 ± 17.24 (31.49,33.72)	0.55 ± 0.24* (0.53,0.56)	3.98 ± 2.01* (3.86,4.10)

*Compared to age group 20~, *p* < 0.05 by Mann–Whitney U test

As shown in Table 1, compared to age 20 ~ 24 group (*n* = 20, 17.3 ± 4.95 nmol/L), serum T level of age 35 ~ 39 (*n* = 101, 14.40 ± 4.33 nmol/L), 40 ~ 44 (*n* = 94, 14.33 ± 4.91 nmol/L), 45 ~ 49 (*n* = 65, 14.22 ± 4.17 nmol/L), 50 ~ 54 (*n* = 63, 14.19 ± 3.93 nmol/L), and 55 ~ 59 (*n* = 87, 14.73 ± 4.87 nmol/L) was significantly decreased (*p* < 0.05). Intriguingly, however, serum T level of age 60 or older did not significantly reduced compared to the age of 20 ~ 24 group (*p* > 0. 05). In contrast, serum LH level of age 60 ~ 64 (*n* = 116, 6.10 ± 3.56 IU/L), 65 ~ 69 (6.33 ± 3.06 IU/L), 70 ~ 74 (6.87 ± 3.86 IU/L), 75 ~ (8.32 ± 4.19 IU/L) was significantly higher than that of age 20 ~ 24 (*n* = 40, 4.06 ± 1.73 IU/L, *p* < 0.05), while serum LH level of age 25 ~ 49 was not significantly increased compared to that of age 20 ~ 24 group (*p* > 0.05). Similar to LH, serum level of SHBG gradually increased with aging and significantly higher from age 45 ~ compared to that of age 20 ~ 24 (*p* < 0.05). FTI was significantly lower after 30 or older (*p* < 0.05) and TSI decreased significantly with aging after 50 or older (*p* < 0.05).

**Fig. 1:**
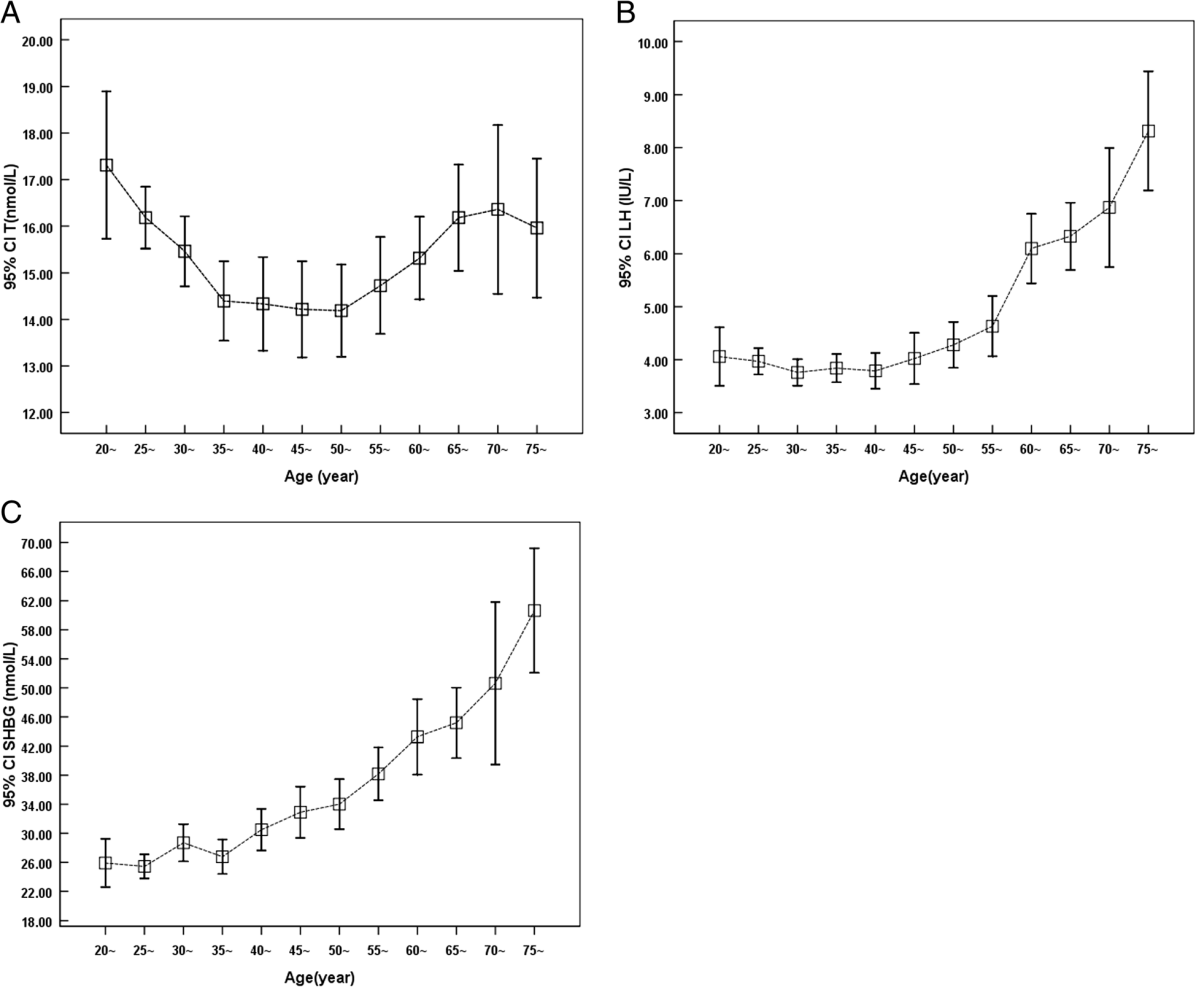
Level of serum testosterone (T), luteinizing hormone (LH), and sex hormone binding globulin (SHBG) with aging. Serum concentration of testosterone (Panel **a**), LH (Panel **b**), and SHBG (Panel **c**) were quantified as described in the method. Vertical axis: mean and 95 % CI; horizontal axis: age groups by 5-year apart

**Fig. 2:**
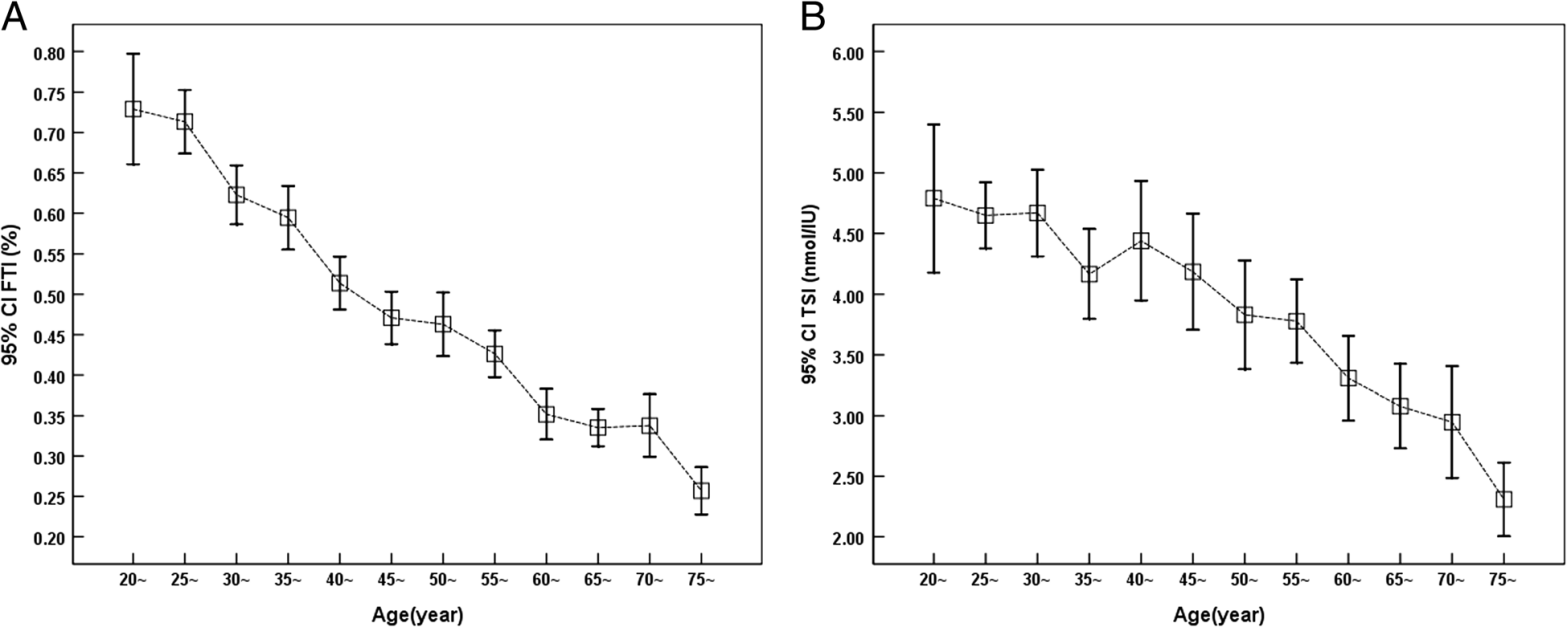
Free testosterone index (FTI) and testosterone secretion index (TSI) with aging. FTI and TSI were calculated as described in the method and presented in Panel **a** and Panel **b**, respectively. Vertical axes: mean and 95 % CI; horizontal axis: age groups by 5-year apart

Serum testosterone gradually decreased till 35 years old followed a plateau at age 35 ~ 50, and a slight increase of serum T was observed between age of 50 ~ 70 years old (Fig. 1a). Serum LH was low at younger age groups (20 ~ 50 years old) followed by gradual increase with aging, and reached a highest level at age of 75 or older (Fig. 1b). Similarly, serum SHBG also gradually increased with aging, reached highest at age of 75 or older (Fig. 1c). In contrast, FTI and TSI were both gradually decreased with aging (Fig. 2a and b, respectively).

### Correlation analysis

The association between sex steroids and the participants’ basic features including age, body mass index (BMI), diphosphoglycerate (BPG), triglyceride (TG), and total cholesterol (TC) was assessed by Spearman rank correlation analysis (Table 2). There was no significant correlation between testosterone and age (*r* = − 0.013, *p* = 0.657) or testosterone level and TC level (*r* = 0.249, *p* = 0.142). However, LH and SHBG were positively correlated with age (*r* = 0.422, 0.475,respectively, *p* < 0. 001), and in contrast, FTI and TSI were negatively correlated with age (*r* = −0.342, −0.569,respectively, *p* < 0.001). Intriguingly, BMI was inversely and significantly correlated with levels of testosterone (*r* = −0.585, *p* < 0.001), LH (*r* = −0.090, *p* < 0.001), SHBG (*r* = − 1.817, *p* < 0.001), and TSI (*r* = − 0.104, *p* < 0.001), but positively and significantly correlated with FTI level (*r* = 0.011, *p* < 0.001). In addition, T and SHBG were negatively and significantly correlated with TG (*p* < 0.001), but not with TC (*p* > 0.05). FTI was significantly correlated with TG (*p* < 0.001), but negatively correlated with TC (*p* = 0.028).

**Table 2:** Correlations between basic features and levels of T, LH, SHBG, FTI, and TSI in chinese male

	Statistical value*	T	LH	SHBG	FTI	TSI
Age	r	−0.039	0.369	0.421	−0.641	−0.375
	*p*	0.195	0.000	0.000	0.000	0.000
BMI	r	−0.585	−0.090	−1.817	0.011	−0.104
	*p*	0.000	0.000	0.000	0.000	0.000
BPG	r	−0.332	−0.035	−0.612	−0.006	−0.059
	*p*	0.027	0.664	0.196	0.344	0.383
TG	r	−0.682	−0.126	−3.087	0.033	−0.103
	*p*	0.000	0.082	0.000	0.000	0.093
TC	r	0.249	−0.212	0.871	−0.017	0.213
	*p*	0.142	0.019	0.102	0.028	0.005

*Spearman’s Correlation Coefficient. *T* testosterone, *LH* luteinizing hormone (LH), *SHBG* sex hormone binding globulin, *FTI* free testosterone index, *TSI* testosterone secretion index, *BMI* body mass index, *BPG* diphosphoglycerate, *TG* triglyceride, *TC* total cholesterol

## Discussion

This study was designed to determine dynamic alteration of testosterone in healthy Chinese male by a single-center population-based cross-sectional study. Serum level of total testosterone (T), luteinizing hormone (LH), and sex hormone binding globulin (SHBG) were quantified. Testosterone secretion index (TSI) and free testosterone index (FTI) were then calculated based on total T, LH and SHBG. It was found that average serum total T concentration was 15.36 ± 4.86 nmol/L, LH was 4.76 ± 2.76 IU/L, and SHBG was 32.61 ± 17.24 nmol/L. Calculated mean FTI was 0.55 ± 0.24, and mean TSI was 3.98 ± 2.01 nmol/IU.

Testosterone is the major androgen in male, and 95 % testosterone is secreted by the Leydig cells and 5 % is secreted by the adrenal glands. Adult Leydig cells are terminally differentiated cells, and secretion of testosterone (T) by Leydig cells is regulated by luteinizing hormone (LH), which is synthesized and produced by the pituitary gland. The secretion of T is regulated by the hypothalamic-pituitary-gonadal axis. Approximately 98 % of circulating testosterone is bound to plasma proteins, with the remaining 2 % circulating freely.

Many studies have now demonstrated that as men aging, their serum testosterone concentrations fall. These studies include both cross-sectional and longitudinal studies. In this context, in the Baltimore Longitudinal Study of Aging, serum total testosterone concentration decreased modestly with increasing age, and an index of the free testosterone concentration decreased even more [18]. Longitudinal observation in the Massachusetts Male Aging Study also showed a decrease in total testosterone with increasing age and a greater decrease in free testosterone [19]. Feldman HA [20] reported that total T declined cross-sectionally at 0.8 %/yr of age, both free and albumin-bound T declined at about 2 %/yr, sex hormone-binding globulin increased cross-sectionally at 1.6 %/yr; that the longitudinal decline in total T was 1.6 %/yr and bioavailable T was 2–3 %/yr. The mechanisms of testosterone reduction with aging seem to reflect changes at all levels of the hypothalamic-pituitary-testicular axis [21]. In this regard, gonadotrophin levels rose during aging [20] and testicular secretory responses to recombinant human chorionic gonadotrophin (hCG) were reduced [22], which implies that reduced production of testosterone may be caused by primary testicular failure. In addition, change in the lutenising hormone (LH) production may also contribute to the reduction of testosterone in aged population [22, 23].

In the current study, interestingly, level of the total testosterone showed a decreasing tendency from age of 20 to 35 years old followed by a plateau at 35 ~ 50, and then an increasing tendency after age of 50 and reached highest at age 70. The LH was lower in the age of 20s and 30s, followed by a gradual increase at 40s, and significant increase after 50s, especially at 60s and 70s age group. The SHBG showed an increase with aging, and in contrast, FTI and TSI were reduced with aging. The appearance of plateau at age 35 ~ 50 and increasing tendency after age of 50 in testosterone concentration was unexpected. While the mechanism of this phenomenon remains to be defined, it might attribute to the gradual increase of LH after 40s, which was demonstrated in the current study.

While many published data showed that serum testosterone decreased with aging [6, 24], studies also indicated that serum total testosterone were not significantly altered in aged population compared to young population. In this context, Frost M [25] investigated 783 men aged 20–29 years and 600 men aged 60–74 years who were randomly recruited from the background population, and found that reference intervals of total T (TT) in healthy young men (11.7–37.7 nmol/l) were comparable to those observed in healthy, elderly men (11.2–37.8 nmol/l), but reference intervals for free testosterone (FT) and bioavailable testosterone (BT) were lower in elderly men due to higher levels of SHBG. Yeap et al. [26] found that TT did not decline with advancing age (aged 70–89, *n* = 3645). Halmenschlager et al. [27] (*n* =428) reported not only was decreased in TT with advancing years, but also was increased in variance later in life. Frost et al. [25], Boyce et al. [28] and Orwoll et al. [29] (*n* = 783, 266, 2,623 respectively) reported not only was there no decline in serum TT with advancing age but also no increase in variance. Rhoden et al. [30] reported that not only did serum TT not fall with advancing age, but there was also an increase in variance across the lifespan from age 40 onwards (*n* =1,071). Kelsey TW et al. [31] analyzed a dataset obtained from 13 studies (*n* = 10,097; age range 3–101 years), and found no evidence to support a progressive decline in testosterone in middle aged and older men.

Consistently, the current study also found that serum total testosterone levels were relatively stable with slight fluctuation between the age groups by 5-year apart from 20 ~ 87 years old, and that correlation analysis indicated that there was no correlation between age and total testosterone level. In contrast, BMI was inversely and significantly correlated with serum T level, suggesting that measurement of total T should be interpreted in concert with factors such as an assessment of health status and body composition rather than simply age. Relative stable status of total testosterone found by us and many other investigators may be due to a decline in the number and ability of Leydig cells leading to gradual decline of the serum androgen levels, which in turn renders the hypothalamic pituitary secrete more LH and results in stimulating T synthesis by tests. In addition, along with the increase of age, the serum LH and SHBG levels gradually increase, which may contribute to gradual decline of FTI and TSI.

Correlation between body mass index (BMI) and serum level of total and free testosterone in healthy men has been reported by Diaz-Arjonilla et al. [5] and it was found that the serum total and free testosterone was significantly and inversely related to increasing BMI (*r* = − 0.32, *p* < 0.001 for total; *r* = − 0.45, *p* < 0.003 for free testosterone). Consistent with this report, while aging was not significantly correlated with the alteration of total testosterone level in the current study, body mass index (BMI) was negatively and significantly correlated to the levels of testosterone, LH, SHBG, and TSI. In addition, BPG and TG, but not TC, were significantly and negatively correlated to total T level. While the mechanism of negative correlation of BMI and total testosterone remains to be defined, it has been explained, at least in part, by lower sex hormone-binding globulin (SHBG) in obsess men [4]. Studies also indicated that the relationship between androgens and BMI is likely bi-directional. In this content, on one hand, morbid obesity has negative effects on the hypothalamic-pituitary-gonadal axis in men [32], and on the other hand, low testosterone levels predispose to central and visceral adiposity [33, 34]. These findings suggest that body weight may be a key contributor in modulating serum testosterone level.

Taken together, the current study demonstrated that serum total testosterone level was relatively stable with aging in healthy Chinese male; level of LH and SHBG gradually increased with aging and were correlated with age; while free testosterone index (FTI) and testosterone secretion index (TSI) gradually decreased with aging and were negatively correlated with age. While aging was not significantly related to serum level of total testosterone, body mass index was significantly and inversely correlated to the serum testosterone level.
